# Earthworm activity optimized the rhizosphere bacterial community structure and further alleviated the yield loss in continuous cropping lily (*Lilium lancifolium* Thunb.)

**DOI:** 10.1038/s41598-021-99597-y

**Published:** 2021-10-21

**Authors:** Yaoxiong Lu, Peng Gao, Yunsheng Wang, Wei Li, Xinwei Cui, Jiamin Zhou, Fuyuan Peng, Liangying Dai

**Affiliations:** 1 College of Plant Protection, Hunan Agricultural University, Changsha, 410128 China; 2 Institute of Agro-Environment and Ecology, Hunan Academy of Agricultural Sciences, Changsha, 410125 China

**Keywords:** Microbial ecology, Microbial communities, Biotic

## Abstract

The soil microbial community plays a vital role in the biogeochemical cycles of bioelements and maintaining healthy soil conditions in agricultural ecosystems. However, how the soil microbial community responds to mitigation measures for continuous cropping obstacles remains largely unknown. Here we examined the impact of quicklime (QL), chemical fungicide (CF), inoculation with earthworm (IE), and a biocontrol agent (BA) on the soil microbial community structure, and the effects toward alleviating crop yield decline in lily. High-throughput sequencing of the 16S rRNA gene from the lily rhizosphere after 3 years of continuous cropping was performed using the Illumina MiSeq platform. The results showed that *Proteobacteria*, *Acidobacteria*, *Bacteroidetes*, *Actinobacteria*, *Chloroflexi* and *Gemmatimonadetes* were the dominant bacterial phyla, with a total relative abundance of 86.15–91.59%. On the other hand, *Betaproteobacteriales*, *Rhizobiales*, *Myxococcales*, *Gemmatimonadales*, *Xanthomonadales,* and *Micropepsales* were the dominant orders with a relative abundance of 28.23–37.89%. The hydrogen ion concentration (pH) and available phosphorus (AP) were the key factors affecting the structure and diversity of the bacterial community. The yield of continuous cropping lily with using similar treatments decreased yearly for the leaf blight, but that of IE was significantly (*p* < 0.05) higher than with the other treatments in the same year, which were 17.9%, 18.54%, and 15.69% higher than that of blank control (CK) over 3 years. In addition, IE significantly (*p* < 0.05) increased organic matter (OM), available nitrogen (AN), AP, and available potassium (AK) content in the lily rhizosphere soil, optimized the structure and diversity of the rhizosphere bacterial community, and increased the abundance of several beneficial bacterial taxa, including *Rhizobiales, Myxococcales, Streptomycetales* and *Pseudomonadales.* Therefore, enriching the number of earthworms in fields could effectively optimize the bacterial community structure of the lily rhizosphere soil, promote the circulation and release in soil nutrients and consequently alleviate the loss of continuous cropping lily yield.

## Introduction

Lily is a perennial herb of the *Lilium* genus, monocotyledons subclass. It is widely cultivated in east Asia, Europe and North America benefiting from its high medicinal, edible, and ornamental value^1–3^. Lily cultivation in China has obvious regional characteristics, and has a long planting history. However, this crop is usually cropped for several years on the same arable land^4^, which will changes physical and chemical soil properties, resulting in self-toxic allelochemicals^4,5^. These chemical compounds are usually secreted by the roots or produced by the decomposition of root residues, which tend to cause a direct rhizosphere microorganisms selection^6,7^ and lead to a soil microbial community structure imbalance^8^, to be the main cause of continuous cropping obstacles. These will subsequently result in decreasing of the diversity and richness indices of bacterial community^9^. The main manifestations include increasing in fungal pathogens^10^, and decreasing in beneficial bacteria^11^ and ratio of bacteria to fungi. Consequently, bacteria-type soil shifts to fungi-type soil^10^. In particular, the number of pathogenic microorganisms such as *Fusarium* increases and causes the occurrence of lily leaf blight disease, then leads to a decline in lily yield and quality^9,11,12^.

In order to effectively alleviate the decline in the yield of continuous crop, physical, chemical, and biological methods have been formulated to ameliorate this soil characteristic. Lime and ammonium bicarbonate are usually applied as physical agents to ameliorate acidic or alkaline soil^13^. In contrast chemical methods include the use of trifloxystrobin, carbendazim, mancozeb, thiram and chlorothalonil^14,15^, and biological methods include using biocontrol bacteria^16^, biological organic fertilizer^17^, and earthworm activities (wormcast)^18,19^. All of these treatments are beneficial for optimizing the microbial community structure of continuous cropping soils, and adjust the unfavorable factors affecting plant growth and directly inhibiting the rapid growth of soil fungi. Because the bacterial community and the interactions among different bacterial taxa play essential roles in continuous cropping fields^8^, the composition and structure of the soil bacterial community could be used as a new indicator of soil ecosystem health, productivity and environmental disturbance^20,21^. As an important component of the soil ecosystem, soil bacteria participates in nutrient cycling, organic matter (OM) decomposition, energy conversion^22,23^, and plays an important role in inhibiting soil-borne diseases^24^. Shen et al.^25^ found that an increased abundance of *Pseudomonas* significantly positively correlates with soil available phosphorus (AP) and disease inhibition. Pearson's correlation demonstrated that the abundance of *Arthrobacter* and *Lysobacter*, which are considered to be beneficial bacteria, had a significant negative correlation with bacterial wilt disease^24^.

However, the bacterial community changes with continuous cropping lily rhizosphere remains largely unknown. In this study, the responses of the soil microbial community were investigated for continuous cropping lily (*Lilium lancifolium* Thunb.) for 3 years when the mitigation measures were applied for continuous cropping obstacles. Quicklime, thiram, earthworm inoculation, and a biocontrol agent were used to alleviate the characters of continuous cropping lily. Leaf blight and yield, soil properties, and the rhizosphere bacterial community were investigated to provide a theoretical basis for exploring an efficient cultivation technique for continuous cropping lily.

## Results

### Effects of different treatments on the disease index of leaf blight and lily bulb yields

**Figure 1:**
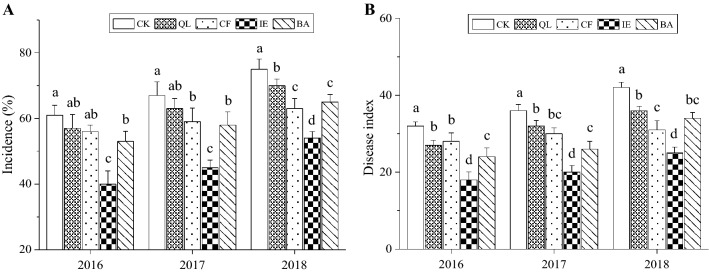
Effects of different treatments on the incidence (**A**) and disease index (**B**) of continuous cropping lily leaf blight. Bars indicate standard error (n = 3). Different letters above columns within the same species indicate significance at p < 0.05 according to Duncan’s test. *CK* blank control, *QL* quicklime, *CF* chemical fungicide, *IE* inoculation with earthworms, *BA* biocontrol agent.

In order to reveal the effects of different treatments on the yield from continuous cropping, lily was planted continuously from 2015 to 2018 and treated with five different ways: blank control (CK), quicklime (QL), chemical fungicide (CF), inoculation with earthworms (IE), and a biocontrol agent (BA). IE significantly reduced the incidence and disease index of lily leaf blight (Fig. 1A,B). The leaf blight incidence was 61%, 67%, and 75% in CK over the 3 consecutive years, and 40%, 45% and 54%, respectively, in IE (Fig. 1A). Furthermore, the disease index was 32, 36, and 42 in CK over the 3 consecutive years, but IE significantly reduced this index (18, 20 and 25 respectively; Fig. 1B). The disease index was 27, 32, and 36 in QL over the 3 consecutive years, 28, 30 and 31 in CF, and 24, 26 and 34 in BA, respectively. The other three treatments also reduced the disease index and incidence, but IE treatment was the best choice in comparison.

**Figure 2:**
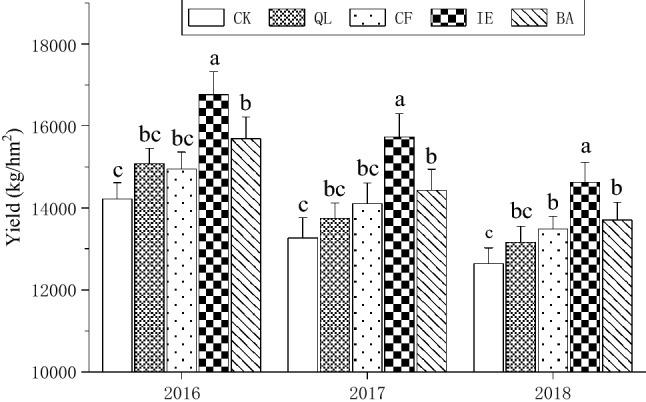
Effects of different treatments on the yield of continuous cropping lily. Bars indicate standard error (n = 3). Different letters above columns within the same species indicate significance at p < 0.05 according to Duncan’s test. *CK* blank control, *QL* quicklime, *CF* chemical fungicide, *IE* inoculation with earthworms, *BA* biocontrol agent.

In this study, different treatments dramatically altered the bulb yield of continuous cropping lily (Fig. 2). A comparison of the same treatment over different years showed that the yield of continuous cropping lily decreased annually. Comparison of different treatments within the same year showed that IE resulted in the highest yield, which was significantly (*p* < 0.05) higher than the other treatments. The yield of continuous cropping lily over the 3 consecutive years for IE was 16,758.13 kg hm^−2^, 15,733.2 kg hm^−2^, and 14,620.65 kg hm^−2^, which is 17.9%, 18.54%, and 15.69% higher than that of CK, respectively. In addition, the bulb yields negatively correlated with disease index and incidence (Fig. S1).

### Effects of different treatments on the availability nutrients of lily rhizosphere soils

**Figure 3:**
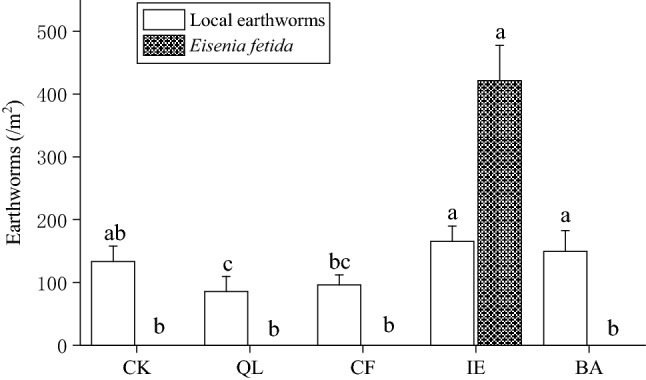
Effects of different treatments on the number of earthworms. Bars indicate standard error (n = 3). Different letters above columns within the same species indicate significance at p < 0.05 according to Duncan’s test. *CK* blank control, *QL* quicklime, *CF* chemical fungicide, *IE* inoculation with earthworms, *BA* biocontrol agent.

The rhizosphere is the soil zone surrounding the plant roots where the features of the soil are influenced by the plants. Our study established that the five different treatments dramatically altered the physical and chemical properties of the rhizosphere during continuous cropping of lily (Table 1). The pH of rhizosphere soil with the QL treatments was 7.2, which was significantly (*p* < 0.05) higher than the other treatments, followed by IE (6.02), with the lowest pH observed in CK (5.27). The soil OM (30.25 g kg^−1^), total nitrogen (TN, 2.07 g kg^−1^), alkali-hydrolyzable nitrogen (AN, 129.41 mg kg^−1^), AP (104.93 mg kg^−1^), and available potassium (AK, 184.99 mg kg^−1^) were highest in the IE treated of the continuous cropping lily rhizosphere. The OM, AN, AP and AK were significantly (p < 0.05) higher with IE than the other treatments. Total phosphorus (TP, 1.22 g kg^−1^) and total potassium (TK, 26.68 g kg^−1^) were the highest in CF and significantly (*p* < 0.05) higher than other treatments. There were eight species of native earthworms in the five treatments, including one epigeic species (*Amynthas cortices*), four endogeic species (*Amynthas robustus*, *Drawida gisti*, *Drawida japonica*, and *Pheretima pingi*), and three anecic species (*Lumbricus terrestris*, *Metaphire guillelmi*, and *Pheretima guillelmi*)^26,27^. The local earthworms population were lower in QL (85.33 m^−2^) and CF (96 m^−2^; Table 1), because the presence of QL and CF would drives earthworms away. IE treatment had the largest earthworm population (586.67 m^−2^) includes 421.33 m^−2^ exotic *Eisenia fetida* (epigeic species, compost earthworm) and 165.33 m^−2^ native earthworms (Fig. 3), which was significantly (*p* < 0.05) higher than the other treatments and 3.4-times higher than CK (133.33 m^−2^ native earthworms only).

**Table 1:** Effects of different treatments on rhizosphere soil properties in continuous cropping lily.

Sample code	pH	OM (g kg^−1^)	TN (g kg^−1^)	TP (g kg^−1^)	TK (g kg^−1^)	AN (mg kg^−1^)	AP (mg kg^−1^)	AK (mg kg^−1^)	Earthworms (m^−2^)
CK	5.27 ± 0.07d	27.66 ± 0.48bc	1.89 ± 0.05bc	1.16 ± 0.04b	24.81 ± 0.80b	92.29 ± 6.28d	74.71 ± 7.49c	103.24 ± 6.56d	133.33 ± 24.44bc
QL	7.20 ± 0.10a	26.63 ± 0.53c	1.84 ± 0.06c	1.09 ± 0.03c	24.59 ± 1.04b	97.56 ± 6.31 cd	56.05 ± 6.17d	138.94 ± 7.51c	85.33 ± 24.44c
CF	5.36 ± 0.13d	27.04 ± 0.77c	2.03 ± 0.06a	1.22 ± 0.06a	26.68 ± 0.58a	113.72 ± 5.74b	87.92 ± 4.41b	160.95 ± 6.18b	96.00 ± 16.00c
IE	6.02 ± 0.10b	30.25 ± 1.04a	2.07 ± 0.06a	1.04 ± 0.03c	23.84 ± 0.60b	129.41 ± 7.83a	104.93 ± 9.31a	184.99 ± 5.34a	586.67 ± 66.61a
BA	5.63 ± 0.09c	28.55 ± 0.69b	1.93 ± 0.04b	1.07 ± 0.05c	24.39 ± 0.89b	112.60 ± 10.15bc	85.77 ± 4.69b	170.77 ± 5.56b	149.33 ± 33.31b

Date are presented as the Mean ± Standard Deviation (n = 3). Different letters within columns followed by indicate significance at *p* < 0.05 according to Duncan’s test.

*CK* blank control, *QL* quicklime, *CF* chemical fungicide, *IE* inoculation with earthworms, *BA* biocontrol agent, *pH* hydrogenion concentration, *OM* soil organic matter, *TN* total nitrogen, *TP* total phosphorus, *TK* total potassium, *AN* alkali-hydrolyzable nitrogen, *AP* available phosphorus, *AK* available potassium.

### High-throughput sequencing of microbial community from lily rhizosphere soil

The plant rhizosphere has been reported to harbor many unculturable microorganisms that benefit crop production. In this study, the Illumina MiSeq platform was used to sequence the V3-V4 hypervariable regions of the bacterial 16S rRNA gene. A total of 788,539 reads (52,569 ± 6280 per sample) with an average length of 469.74 ± 2.33 bp were obtained from the 15 rhizosphere samples of continuous cropping lily under the five different treatments. Using a cut-off of 97% sequence similarity, 2521 operational taxonomic units (OTUs) were obtained. After flat screening, 19,790 high-quality sequences were obtained for each sample, including 2093–2231 operational taxa. The values of the Chao1 estimator (2165.78) and Simpson diversity index (44.16 × 10^–4^) were highest in IE (Table 2). The results indicated that IE was beneficial for optimizing the bacterial community structure and diversity of the rhizosphere soil during continuous cropping of lily. The Good’s nonparametric coverage of each sample library ranged from 98.69 to 99.13%, indicating that the OTU coverage of the sample was high enough and represented the real bacterial community of the rhizosphere soil in continuously cropped lily under different treatments.

**Table 2:** Bacterial α–diversity of rhizosphere soil in continuous cropping lily.

Sample code	Chao1	Simpson (× 10^–4^)	Coverage (%)
CK	2164.97 ± 71.61a	24.59 ± 3.99a	99.13 ± 0.09a
QL	2016.79 ± 51.42b	34.93 ± 6.41a	98.84 ± 0.14a
CF	2104.03 ± 47.11ab	37.16 ± 12.83a	98.99 ± 0.14a
IE	2165.78 ± 87.94a	44.16 ± 24.08a	98.69 ± 0.63a
BA	2011.51 ± 86.18b	31.48 ± 1.40a	98.94 ± 0.18a

Date are presented as the Mean ± Standard Deviation (n = 3). Different letters within columns followed by indicate significance at *p* < 0.05 according to Duncan’s test.

*CK* blank control, *QL* quicklime, *CF* chemical fungicide, *IE* inoculation with earthworms, *BA* biocontrol agent.

**Figure 4:**
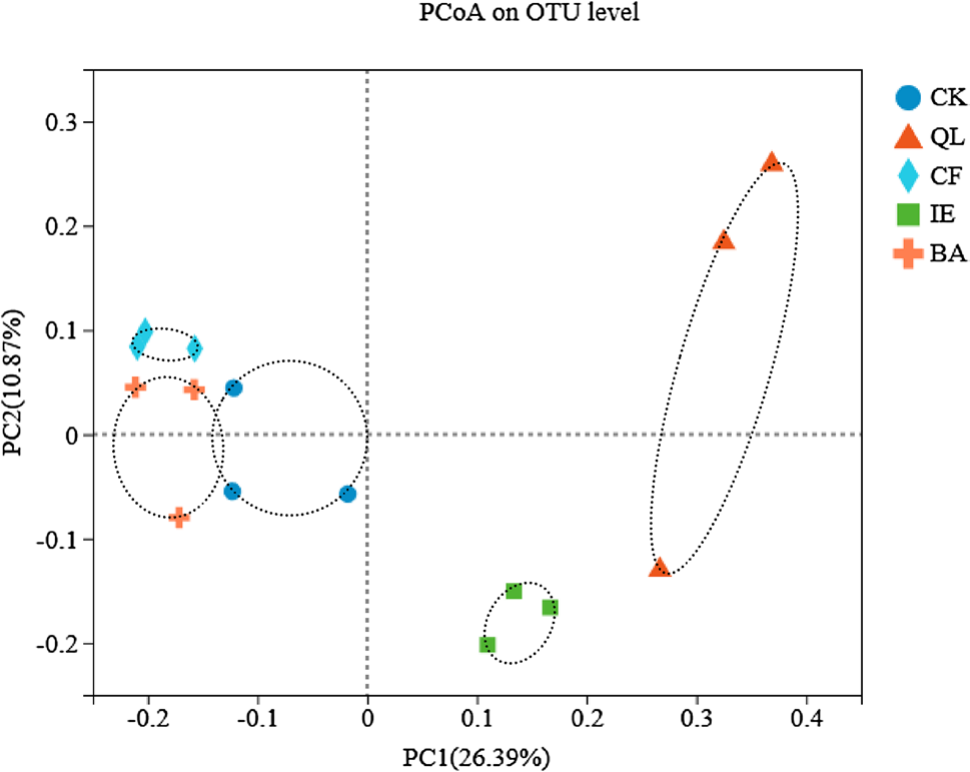
PCoA of bacterial community of the rhizosphere soil in continuous cropping lily. *CK* blank control, *QL* quicklime, *CF* chemical fungicide, *IE* inoculation with earthworms, *BA* biocontrol agent.

Principal coordinates analysis (PCoA) was performed based on the OTU level (Fig. 4). The multi-dimensional rhizosphere soil bacterial variables were reduced to two, with the first (PC1) and second (PC2) principal coordinates contributing to 26.39% and 10.87% of the variation, respectively, representing a cumulative contribution rate of 37.2%. There was a clear separation between different treatments, and an apparent aggregation among rhizosphere soil samples from the same treatment, indicating that the difference in microbial diversity was more significant between groups than within the group.

### Composition of bacterial community at the phylum and order levels in lily rhizosphere soil

**Figure 5:**
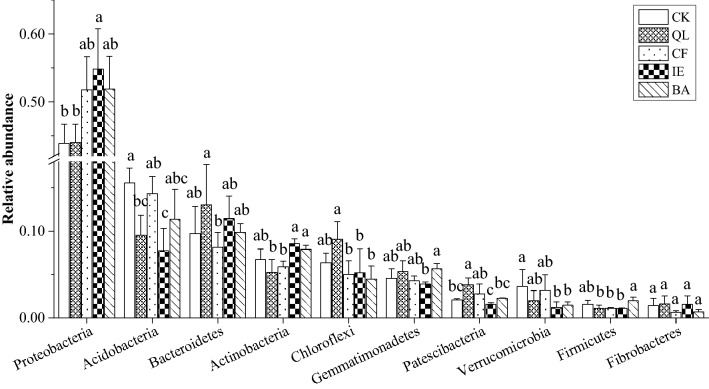
Relative abundance of the top ten dominant bacterial phyla of rhizosphere soil in continuous cropping lily. Bars indicate standard error (n = 3). Different letters above columns within the same species indicate significance at p < 0.05 according to Duncan test. *CK* blank control, *QL* quicklime, *CF* chemical fungicide, *IE* inoculation with earthworms, *BA* biocontrol agent.

At the phylum level, 33 taxonomic groups were identified. Taxonomic groups with an average relative abundance < 1% were classified as other, resulting in 12 phyla. *Proteobacteria* (43.86–54.80%), *Acidobacteria* (7.70–15.55%), *Bacteroidetes* (8.11–13.02%), *Actinobacteria* (5.23–8.56%), *Chloroflexi* (4.45–9.05%), and *Gemmatimonadetes* (3.88–5.64%) were the dominant bacterial phyla, corresponding to a total relative abundance of 86.15–91.59% (Fig. S2). Compared to the other treatments, IE had the highest abundance of the phyla *Proteobacteria* (54.80%) and *Actinobacteria* (8.56%). The relative abundance of *Proteobacteria* was significantly (*p* < 0.05) higher than that of CK (43.86%), and the relative abundance of *Actinobacteria* being significantly (*p* < 0.05) higher than that of QL (5.23%). However, IE had the lowest relative abundance for the phyla *Acidobacteria* (7.70%)*, Gemmatimonadetes* (3.88%)*, Patescibacteria* (1.53%)*,* and *Verrucomicrobia* (1.16%), with the abundance of *Acidobacteria* significantly (*p* < 0.05) lower than that of CK (15.55%), with a decrease of 50.47% (Fig. 5).

**Figure 6:**
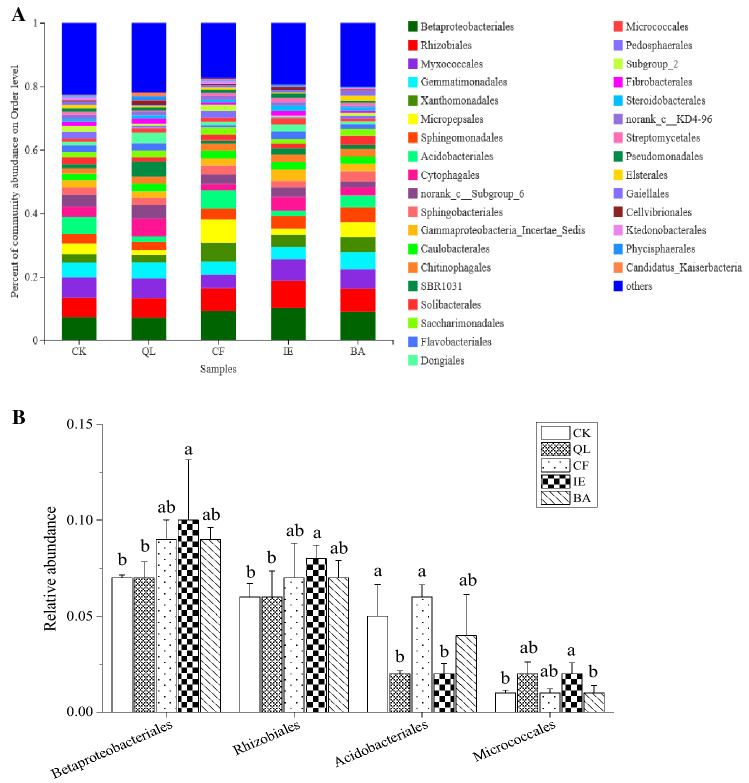
Relative abundance of bacterial orders of rhizosphere soil in continuous cropping lily. (**A**) The relative abundances of the bacterial orders of continuous cropping lily, and the taxonomic groups with an average relative abundance of < 1% were classified as other. (**B**) The relative abundances of four bacterial orders that were significantly higher or lower than the blank control (CK). *QL* quicklime, *CF* chemical fungicide, *IE* inoculation with earthworms, *BA* biocontrol agent.

The rhizosphere bacteria were further classified at the order level resulting in 193 taxonomic groups. Taxonomic groups with an average relative abundance < 1% were classified as other, resulting in 33 orders (Fig. 6A). Among the resulting 33 orders, two were of middle rank genealogy in the taxonomic database, with no scientific name and marked as Norank. *Betaproteobacteriales* (6.92–10.22%), *Rhizobiales* (6.26–8.38%), *Myxococcales* (4.21–6.97%), *Gemmatimonadales* (3.81–4.48%), *Xanthomonadales* (2.37–5.78%) and *Micropepsales* (1.45–7.34%) were the dominant bacterial orders of the rhizosphere, corresponding to a total relative abundance of 28.23–37.89%. Compared to the other treatments, IE had the highest relative abundance (> 1%) of the orders of *Betaproteobacteriales* (10.22%)*, Rhizobiales* (8.38%)*, Myxococcales* (6.97%)*, Gammaproteobacteria_Incertae_Sedis* (3.55%)*, Flavobacteriales* (2.42%)*, Micrococcales* (2.09%)*, Steroidobacterales* (1.64%)*, Streptomycetales* (1.44%), and *Pseudomonadales* (1.28%)*,* with a significantly (p < 0.05) higher abundance of *Betaproteobacteriales, Rhizobiales,* and *Micrococcales* than with CK (7.11%, 6.26%, and 0.99%, respectively; Fig. 6B; Fig. S3). However, IE had the lowest relative abundance (> 1%) for the orders *Gemmatimonadales* (3.81%), *Acidobacteriales* (1.61%)*, Sphingobacteriales* (2.01%) and *Saccharimonadales* (1.31%), with a significantly lower abundance of *Acidobacteriales* (*p* < 0.05) than with CK (5.24%; Fig. 6B).

### Relationships between lily rhizosphere soil properties and bacterial community

**Figure 7:**
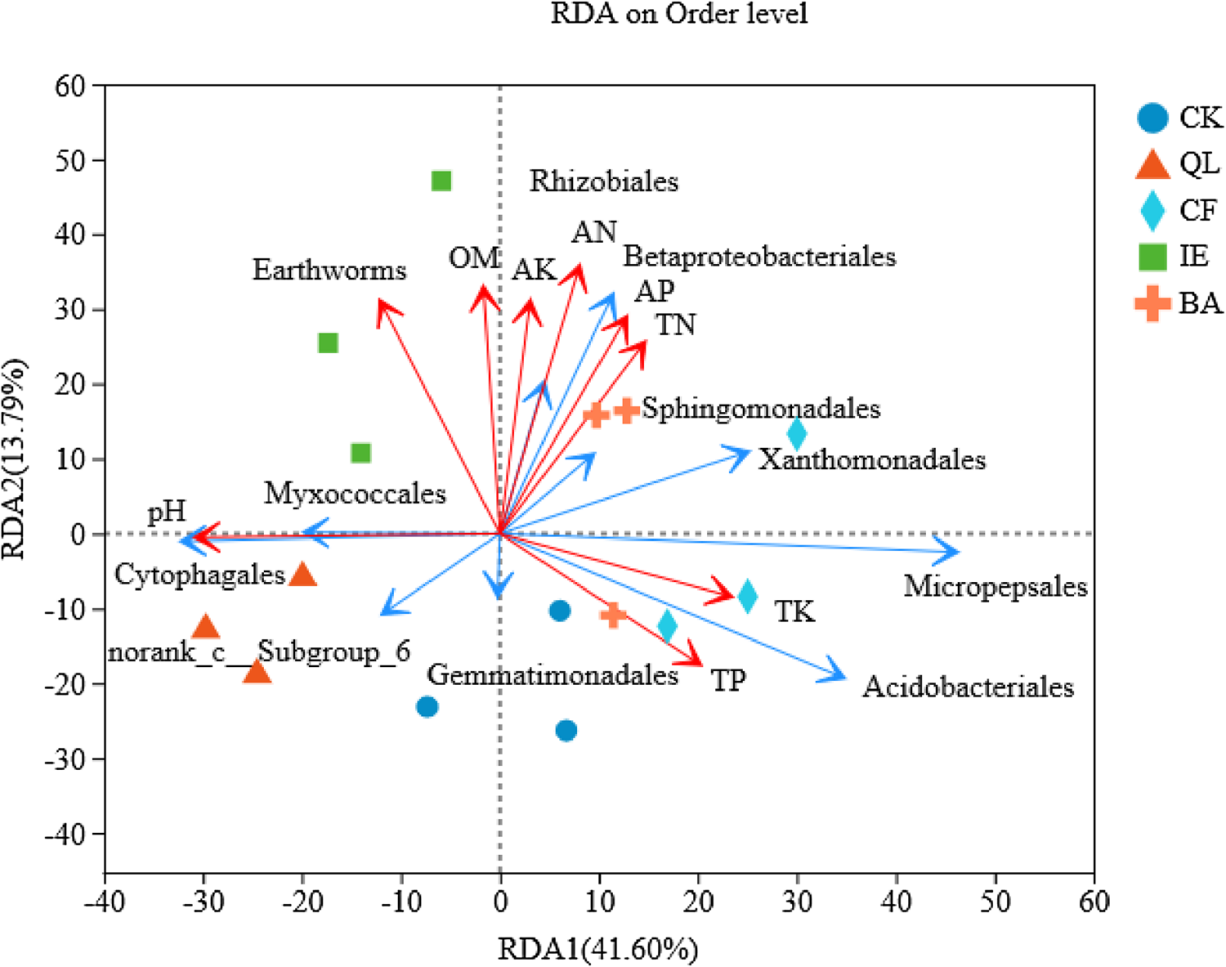
Redundancy analyses of the correlations between rhizosphere soil properties and bacterial community. The blue arrows represent different bacterial orders. The red arrows represent different environmental factors, the length of the arrow of environmental factors represent the degree of environmental factors' influence on the species data (the amount of interpretation). *CK* blank control, *QL* quicklime, *CF* chemical fungicide, *IE* inoculation with earthworms, *BA* biocontrol agent.

To investigate correlations between rhizosphere soil properties and the bacterial community of continuous cropping lily under different treatments, redundancy analysis (RDA) was performed at the order level (Fig. 7). The results showed that the cumulative amount of explained variation in the first (41.6%) and second (13.79%) axes reached 55.39%. *Betaproteobacteriales, Sphingomonadales* and *Xanthomonadales* were significantly (*p* < 0.05) positively correlated with TN, AN, AP and AK. *Rhizobiales* were significantly (*p* < 0.05) positively correlated with OM, TN, AN and AP. *Micropepsales* were significantly (*p* < 0.05) positively correlated with TP and TK, and significantly (*p* < 0.05) negatively correlated with pH. *Acidobacteriales* were significantly (*p* < 0.05) negatively correlated with pH. *Cytophagales* were significantly (*p* < 0.05) positively correlated with pH, and significantly (*p* < 0.05) negatively correlated with TP and TK (Fig. S4).

Furthermore, the Mantel test was employed to investigate Spearman correlations between the relative abundances of bacterial orders and the rhizosphere soil properties of continuous cropping lily (Table 3). The results showed that pH (r = 0.8007) and AP (r = 0.5804) significantly (*p* < 0.01) correlated with the rhizosphere bacterial community composition of the soil of continuous cropping lily. However, OM, TN, TP, TK, AN, AK, and earthworms slightly affected the bacterial community composition. This indicates that pH and AP were the key factors affecting the bacterial community structure at the order level in the rhizosphere of continuous cropping lily.

**Table 3:** Spearman correlations (r) between the relative abundances of bacterial orders and the environmental variables determined by the Mantel test.

Environmental variable	r	*p*
pH	0.8007	0.001
OM	0.793	0.614
TN	0.1337	0.348
TP	− 0.1453	0.293
TK	− 0.0676	0.618
AN	0.0544	0.688
AP	0.5804	0.001
AK	0.0350	0.799
Earthworms	0.0872	0.558

*pH* hydrogen ion concentration, *OM* soil organic matter, *TN* total nitrogen, *TP* total phosphorus, *TK* total potassium, *AN* alkali-hydrolyzable nitrogen, *AP* available phosphorus, *AK* available potassium.

**Figure 8:**
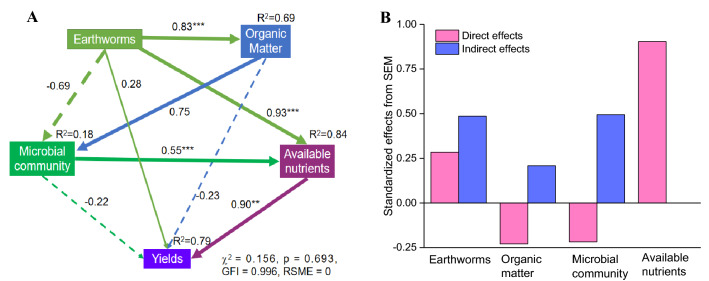
The structural equation model (SEM) between environmental factors and lily yield (**A**), and the direct and indirect effects of environmental variables on lily yield (**B**). The width of the arrows in (**A**) indicates the strength of the standardized path coefficient. The solid lines indicate positive path coefficients and dashed lines indicate negative path coefficients, R^2^ values represent the proportion of the variance explained for each endogenous variable. Available nutrients were represented by the first component from a principal-component analysis (PCA) conducted with available nitrogen (AN), available phosphorus (AP), and available potassium (AK). Microbial community is represented by the first component of the PCA of dominant bacteria at order level (the top ten most abundant orders).

According to the structural equation model (SEM), lily yields were mainly affected by available nutrients, which was the first component of the principal-component analysis (PCA) conducted with AN, AP, and AK (Fig. 8). The earthworm, OM content, and microbial community (represented by the dominant bacteria) also have direct and indirect effects on the lily yields. This model could explain 79% of the variance in lily yields.

## Discussion

### Continuous cropping affects lily leaf blight and bulbs yields

Continuous cropping obstacles related to the deteriorating of crop growth condition, yield and quality reduction, and aggravation of diseases due to the continuous planting of the same crop in the same plot over many years. Lily can be used as a medicinal plant, vegetable and cut flower variety. This crop is usually planted around September to October each year, and harvested around the July of the next year, so only one crop is cultivated in the same field every year. Restricted to farmers' planting habits and land resource, lily cultivation has obvious regional characteristics in China^28^. The economic value results in common continuous cropping, which leads to a severe decline in lily yield and quality^29^. In this study, we used different treatments (QL, CF, IE, and BA) to mitigate the continuous cropping obstacles for lily, demonstrating that all these four treatments increased the output of continuous cropping lily compared to CK. However, the lily yield in the same treatment still decreased annually (Fig. 2), which means that physical, chemical, and biological treatments for continuous cropping lily soil can relieve the declining yield. On the contrary, the incidence and disease index of continuous cropping lily in the same treatment increased annually (Fig. 1A,B), resulting in a negative correlation between the bulb yields and disease index or incidence (Fig. S1). These results suggested that the continuous cropping obstacles of lily were remains obscure. Among different treatments, IE was the most effective one to alleviate the loss of continuous cropping lily yield.

### Earthworms activity affect the soil physical and chemical properties to increase the yield of continuous cropping lily

Earthworms are known as “ecosystem engineers”^30^, and they constitute the largest biomass in soil fauna and play an important role in maintaining the structure and function of soil ecosystem. Earthworms are often used as an important indicator of soil health^31^, The excavation and excretion activities of earthworms can raise soil porosity and the formation of soil aggregate^32,33^, increase soil mineral-N and microbial biomass N^34^, and improve soil water infiltration rate^33^. These have been demonstrated to promote lily growth. Long-term continuous cropping can usually causes soil acidification^11^, and then greatly influence the soil microbial community structure^35^. The decrease of bacterial diversity and the increase of fungi in acidified soil will promote the proliferation of soil-borne pathogens and the occurrence of crop diseases^36^, so the pH was a driver of the microbial community (Fig. 7; Table 3). Literature reported that the number and the biomass of fungi decreased by 50% and by 42%, respectively, when soil pH increase from 4.5 to 7.0^37^. Therefore, alleviating soil acidification is beneficial to reduce the occurrence of continuous cropping lily leaf blight. Earthworms secretions, including calcium carbonate substances and nitrogen substances can increase the pH of acidic soil and alleviate soil acidification^38,39^. The inoculated *E. fetida* were fed with cow dung, which boosted their own reproduction, and increased 22.41-times the original input of *E. fetida* in the soil (the number of earthworms was 421.33 m^−2^ in the inoculum, and the number of *E. fetida* input was 18 m^−2^) (Fig. 3). The activities of earthworms, including digging, swallowing and excreting, can effectively increase soil porosity and promote the transformation of organic matter, subsequently increase the organic matter content in the rhizosphere of lily (Table 1), which will then affect soil microbial community structure and diversity of function^40^, and also retard soil acidification^41^. These are advantageous to soil nutrient cycling^42^, increasing the effectiveness of soil nutrients (such as AN, AP, and AK; Table 1), and the nutrient turnover rate^43,44^. In addition, earthworms can secrete humic acids, which are similar to plant hormones^45^. These compounds could promote the growth of lily roots, facilitate the absorption and utilization of nutrients, which is beneficial to the development of continuous cropping lily, and enhances disease resistance and the yield (Fig. 2).

### Earthworms activity affect the soil microbial community structure and diversity to reduce the disease of continuous cropping lily

Earthworms influence the ecological processes mainly through the soil microbial communities^46,47^. They can directly influence microbial community structure through excavation and digestion^48^, or indirectly influence microbial community structure through changing the soil physical and chemical properties, including soil porosity, aggregate stability, pH, and availability nutrient^49^. Earthworms reduced the number of pathogenic fungi by selectively feeding on fungi in soil, destroying fungal hyphae, and competing with fungi for food resources^50,51^. In particular, the decline in the number of *Fusarium* effectively reduced the occurrence of lily leaf blight (Fig. 1A,B), which is consistent with the results of Bi et al.^17^. The microenvironment of earthworm intestinal tract (high humidity, neutral pH, appropriate C/N) is suitable for microbial reproduction. Because the intestinal mucus of earthworm is an unstable substance with high nitrogen content, which can stimulate the growth and reproduction of bacteria^52^. According to available published researches, it can be deduced that the regulation of microbial community structure by earthworms would have enhanced soil microbial activity and enzyme activity^53,54^, improved soil microecological environment and promoted nutrient cycling, which were conducive to plant root growth and nutrients absorption, and improved the yield of lily (Fig. 2). The soil structure and function regulation are mainly because of the close relationship between earthworms and soil microorganisms^55^. Earthworms influenced the abundance and diversity of the rhizosphere bacterial community through their activities and wormcast, which resulted in the apparent aggregation of the rhizosphere bacterial community among different replicates in IE treatment (Fig. 4). IE mainly reduced the bacterial community diversity (Simpson Index, Table 2) and an increase in the abundance of some important bacterial species (Figs. 5, 6A,B), which are consistent with the results of Jayasinghe et al.^56^. At the phylum level, IE increased the relative abundance of *Proteobacteria* and *Actinobacteria*, but reduced the relative abundance of *Acidobacteria*, compared to the other treatments. Previous studies suggest that members of *Proteobacteria* are eutrophic bacteria and positively correlate with soil nutrients^57^. *Actinobacteria* play key roles in OM decomposition and humus formation processes^58,59^. The relative abundance of *Streptomycetales* (phylum *Actinobacteria*) was higher in IE than in the other treatments (Fig. 6A,B), and these taxon can produce a variety of antibiotics (secondary metabolites)^60^ to protect roots from other pathogenic microorganisms^61^. The activity of earthworms can enhance soil porosity and aeration, promote the growth of aerobic bacteria, and reduce anaerobic bacteria (e.g., *Acidobacteriales*). In this study, the relative abundance of *Acidobacteriales* (phylum *Acidobacteria*) significantly negatively correlated with soil pH (Fig. S4), which is in line with the results of Jones et al.^62^. Because the IE treatment increased the number of earthworms, more earthworms dug in the soil and more wormcast was excreted, which then heightened OM diffusion, reduced the soil bulk density, increased the soil porosity and permeability, promoted the growth and reproduction of aerobic microorganisms, and affected the reproduction of anaerobic *Acidobacteriales*. This then led to a lower relative abundance of *Acidobacteriales* in the rhizosphere of IE treated soil compared to the other treatments (Fig. 6A,B) and raised the pH (Table 3). Furthermore, the relative abundance of *Rhizobiales* was the highest in the IE group (Fig. 6A,B). Liu et al*.*^63^ reported a symbiotic relationship between lilies and *Rhizobiales*, which plays an important role in nitrogen fixation, increasing the content of TN and AN in the rhizosphere of continuous cropping lily. The increase in *Flavobacteriales, Streptomycetales* and *Pseudomonadales* by IE treatment should activate the fixed phosphorus and then increase the AP content in the rhizosphere^64^ (Table 1). Besides, the higher abundance of *Myxococcales* in IE-treated soil enhanced the resistance to soil-borne pathogens^65^. Simultaneously, *Pseudomonadales* produced antibacterial metabolites that can effectively inhibit the occurrence of soil-borne pathogens^66^ from diseases in continuous cropping lily. These results suggest that the increased abundance of these beneficial bacteria in IE treatment is helpful by promoting nutrient cycling and improving stress resistance in the rhizosphere^67^, positively affecting the growth and yield of continuous cropping lily, and alleviating the decline in yield.

### Relationship among earthworms and continuous cropping lily

According to the feeding habits and life in different soil depths, earthworms can be divided into three ecological classes, including the epigeic species, the endogeic species, and the anecic species^68,69^. The application of exotic *E. fetida* (epigeic species) was beneficial to observe their abundance and cow dung conversion efficiency under the same organic material conditions, and distinguish the growth and reproduction from that of the native earthworms. In this study, local earthworms of the continuous cropping fields included one epigeic species, four endogeic species, and three anecic species. The epigeic species mainly live in surface soil, feeding on humus, with small size, short reproduction cycle, and can move quickly when disturbed. The endogeic species mainly live in the subsurface soil and feed on the soil organic matter. They have larger size and stronger digging capacity, moving slowly when the soil is disturbed. Anecic species have the largest size and strongest dragging capacity. They eat the humus in the surface soil and draw them into the hole in deep soil^70^. In comparison, *E. fetida* (epigeic species) is a compost earthworm, they live in organic matter and have a strong ability to convert fresh manure into vermicompost^53^. There were more *E. fetida* in IE group than the others (Fig. 3), which would promoted the degradation and diffusion of the organic matter by digging and excreting in the surface soil, and provide an additional food resource for the endogeic and anecic species (Table 1), and stimulated the activity of them^71^. Subsequently, more and more earthworms activity further improved the physical and chemical properties of the underlying soil (Table 1), provided available nutrient for lily growth, optimized the bulb roots rhizosphere bacterial community (Fig. 6A,B), and also alleviated the yield loss of continuous cropping lily. At the same time, the stem roots of lily usually grow in the surface soil, where *E. fetida* active (IE). The surface-applied cow manure provided a food source for *E. fetida* and therefore improved the physical and chemical properties of the surface soil, also provides available nutrient for lily growth, and better increased the yield of continuous cropping lily. Compared to *E. fetida* of IE, local earthworms in the other trentments weakly transform the surface cow manure to soil organic matter and available nutrients (OM, AN, AP, AK; Table 1), causing that the nutrients of cow manure were not easily absorbed and utilized by lily. However, the rhizosphere fungal community, especially the pathogenic fungi (e.g., *Fusarium*, *Alternaria*) and beneficial fungi (e.g., *Mycorrhiza*, *Trichoderma*) should also play crucial roles in alleviating the decline in yield of continuous cropping lily*.* Therefore, whether earthworm inoculation affects the diversity and composition of rhizosphere fungal communities should be investigated in the future.

## Conclusion

Earthworms inoculation (IE) significantly increased the yield of continuous cropping lily, and alleviated the decline in yield. Because IE significantly increased the content of OM, AN, AP, and AK in the rhizosphere, and the pH, and AP are key factors that influence the structure and diversity of bacterial communities. They optimized the structure and diversity of the bacterial community, improving the abundance of some beneficial bacteria, including members of *Rhizobiales*, *Myxococcales*, *Streptomycetales* and *Pseudomonadales*. These beneficial bacteria are helpful to reduce the occurrence of soil-borne diseases. The exotic earthworms inoculation and additional application of cow dung were helpful to understand the effect of earthworms on relieving the continuous cropping obstacle of lily. The results may guide farmers the application of OM (the food of native earthworms) to enrich the native earthworms in the soil, thereby reducing the barriers to continuous cropping.

## Materials and methods

### Experimental site

The experiment was set-up at the Changsha Research Station for Agricultural & Environmental Monitoring, Institute of Subtropical Agriculture, Chinese Academy of Sciences, Hunan Province, China (28° 33′ N; 113° 19′ E; 370 m). This area has a typical subtropical monsoon climate, with an average annual temperature of 17.2 °C, precipitation of 1350 mm, evaporation of 1420 mm, frost-free days of 274, and sunshine of 1663 h. The cultivated soil is granite red soil.

The alleviation of continuous cropping obstacle experiment was initiated in 2015. At the start of the trial (September 18, 2015), the main physical and chemical properties of the surface soil (0–20 cm) were measured (the native earthworms 11.33 ± 3.06 m^−2^, pH 6.13 ± 0.06, soil OM content 22.51 ± 0.76 g kg^−1^, TN 1.82 ± 0.03 g kg^−1^, AN 75.25 ± 3.34 mg kg^−1^, TP 0.94 ± 0.02 g kg^−1^, AP 40.78 ± 1.74 mg kg^−1^, TK 22.80 ± 0.44 g kg^−1^, and AK 88.59 ± 5.40 mg kg^−1^).

### Experimental design

**Figure 9:**
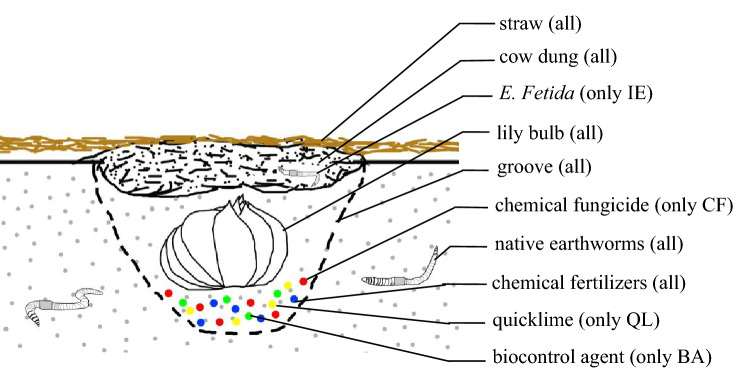
Profile of the lily after planting. The steps for lily planting were as follows. First, every plot was ploughed and ridged. Second, maked grooves in the ridges. Third, applied chemical fertilizer (all the treatments), quicklime (only QL), chemical fungicide (only CF), and biocontrol agent (only BA) at the bottom of the groove, and covered with a small amount of soil. Fourth, putted the lily bulbs in the groove. Fifth, covered the lily bulbs with soil. Sixth, sprinkled cow dung on the lily bulbs. Seventh, inoculated earthworms into cow dung (only IE) and covered the ridges with straw. *QL* quicklime, *CF* chemical fungicide, *IE* inoculation with earthworms, *BA* biocontrol agent.

The completely randomized block field experiment consisted of five treatments: CK, in which were applied only fertilizer and cow dung, but no other artificial treatment was applied; QL, in which 1500 kg hm^**−**2^ of quicklime (CaO) was applied; CF, in which a Thiram wettable powder (68% total effective component content, produced by Hebei Guanlong Agricultural Chemical Co., Ltd, China) was used at a dosage of 15 kg hm^**−**2^ by mixing the fungicide with a small amount of soil and scattering in the groove; IE, in which *Eisenia fetida* was inoculated at 18 m^**−**2^ (75 kg hm^**−**2^ and one earthworm has the weight of 0.40–0.45 g); and BA, in which a powder containing *Bacillus subtilis* (≥ 20 × 10^8^ CFU g^−1^ of total effective viable bacteria; produced by Beijing Qigao Biological Technology Co., Ltd, China) was applied at a dosage of 30 kg hm^**−**2^ by mixing with a small amount of soil and scattering in the groove. Earthworms breathe through their skin and are usually sensitive to external stimuli, so the application of QL and CF will drive them away. Three plot areas (three replicates) were set-up for each treatment. The cow dung was applied at 22,500 kg hm^**−**2^ (according to the dry basis) in each treatment, and the nutrients contained of it (pH, 8.75; OM, 58.86 g kg^−1^; TN, 11.67 g kg^−1^; TP, 3.11 g kg^−1^; and TK 8.50 g kg^−1^) were accounted for the total fertilization calculation to confirm equal nutrition (N, P, K) inputs among different treatments. Full inorganic and organic N, P and K were applied at 450 kg N hm^−2^, 375 kg P_2_O_5_ hm^−2^ and 600 kg K_2_O hm^−2^, respectively, during the lily season. The chemical NPK fertilizers were used as urea (46% N), calcium superphosphate (12% P_2_O_5_), and potassium sulfate (50% K_2_O). First, every plot was ploughed, ridged and grooved (length 1.50 m, width 0.25 m, depth 0.08 m; Fig. 9). Based on the experimental design, QL, CF, chemical fertilizer and BA were deposited at the bottom of the groove and covered by a small amount of soil. Next, lily bulbs were placed in the grooves after disinfection with carbendazim, and then partially covered with soil and sprinkled with fresh cow dung that was the food of *E. fetida*. The earthworms were inoculated according to the dosage in IE. Then covered with a layer of 0.01–0.02 m thick straw on the surfaces of all treatments, to prevent the fresh cow dung from desiccation. The roots system of lily are divided into bulb roots system and stem roots system, the stem roots (about 0–8 cm) and bulb roots (about 8–20 cm) of lily usually grow in the surface soil, where the epigeic and endogeic earthworms are active. Therefore, it is necessary to better understand earthworms activities on the effect of soil bacteria and yield of continuous cropping lily. The row spacing was 0.20 × 0.35 m, and each plot area was 4.50 m^2^ (3.00 m × 1.50 m); the plots were randomly arranged and surrounded by a drainage ditch. The plots inoculated with earthworms were separated by smooth plastic partitions, with 0.30 m of the plastic buried in the soil and 0.50 m of the ground exposed to prevent the earthworms from escaping.

The experimental plots were first planted with lily in 2014, and were designed for continuous cropping of lily from 2015 to 2018 (that is, the plot treatments were fixed after they were designed in 2015). Lily was planted around September 25 of each year, the seedlings emerged in the middle of March of the following year, the flower buds were removed around May 20, and lily bulbs were harvested around July 24.

### Lily disease index of leaf blight and bulb yield

The disease index and incidence of lily leaf blight were investigated at maturity (around 16 July) each year (2016, 2017, and 2018). For each treatment replicate, 50 plants were randomly selected, and the number of infected plant and the number of plants with each disease grade were recorded. The grading standard was defined as follows: 0, no diseased leaves on whole plant; 1, the number of yellow or purple leaves was less than 25%; 2, the number of withered leaves accounted for 25–50%; 3, the leaves of the infected plants were wilted by more than 50%; 4, the whole plant was withered or the plant died. The disease incidence and disease index were calculated according to the following formulas.

$$ {\text{Incidence}} = {\text{number}}\;{\text{of}}\;{\text{infected}}\;{\text{plants}}/{\text{number}}\;{\text{of}}\;{\text{investigated}}\;{\text{plants}} \times 100\%  $$

$$ {\text{Disease}}\;{\text{Index}} = \sum {\left( {{\text{number}}\;{\text{of}}\;{\text{disease-grade}}\;{\text{plants}} \times {\text{the}}\;{\text{representative}}\;{\text{value}}} \right) \times 100\% /\left( {{\text{number}}\;{\text{of}}\;{\text{studied}}\;{\text{plants}} \times {\text{the}}\;{\text{representative}}\;{\text{value}}\;{\text{for}}\;{\text{the}}\;{\text{most}}\;{\text{severe}}\;{\text{disease}}} \right).}  $$

The yield of lily bulbs was measured around 26 July each year, five strains of lily were chosen randomly in each plot and weighed.

### Earthworm, soil sampling, and physicochemical analysis

Earthworms and the lily rhizosphere were sampled during the prosperous stage of lily on April 5, 2018. For the earthworm survey, three soil sections with lily plants in the center (area 0.0625 m^2^, length 0.25 m, width 0.25 m, depth 0.20 m) were randomly selected in each plot to classify *E. fetida* and the local earthworms. Five strains of lily were chosen randomly in each plot. Carefully, the fresh plants were uprooted by a small spade and shaken to separate soil not tightly adhering to the roots^72^. The rhizosphere soil tightly attached to lily bulb roots system was collected. All samples were sealed in separate sterile plastic bags, packed on ice, and immediately transported to the laboratory. Each sample was divided into two subsamples: one subsample was stored at − 80 °C for molecular analysis after freeze-drying. The other was air-dried at room temperature to determine the physical and chemical properties of the soil^73^. Soil pH was measured at a 1:5 ratio of soil to distilled water using a digital pH meter (PHS-3C, INESA Scientific Instrument Co., Ltd, Shanghai, China). OM was assayed via the vitriol acid–potassium dichromate oxidation method. TN was determined according to the semi-micro Kjeldahl method. TP was dissolved using sodium hydroxide and measured using the Mo–Sb colorimetric method. TK was dissolved with sodium hydroxide and measured using the flame photometer method. AN was measured using the alkali-hydrolysis and diffusion method. AP was extracted with sodium bicarbonate and determined via the Mo–Sb colorimetric method. AK was extracted with ammonium acetate and selected using the flame photometer method. All methods were performed in accordance with the relevant guidelines and regulations.

### DNA extraction and PCR amplification

Soil genomic DNA was extracted from 15 samples using the E.Z.N.A. soil DNA Kit (Omega Bio-tek, Norcross, GA, USA) according to the manufacturer’s instructions. The DNA quality was checked on a 1% agarose gel, and the DNA purity was determined using a NanoDrop 2000 UV–Vis spectrophotometer (Thermo Scientific, Wilmington, USA). The V3-V4 hypervariable region of the bacterial 16S rRNA gene was amplified using the universal primers 338F (5′-ACTCCTACGGGAGGCAGCAG-3′) and 806R (5′-GGACTACHVGGGTWTCTAAT-3′) on an ABI GeneAmp 9700 PCR thermocycler (ABI, CA, USA). PCR amplification was performed as follows: initial denaturation at 95 ℃ for 3 min, followed by 27 cycles of denaturation at 95 ℃ for 30 s, annealing at 55 ℃ for 30 s and extension at 72 ℃ for 45 s, and a final extension at 72 ℃ for 10 min. Each PCR mixture contained 4 μL 5× TransStart FastPfu buffer, 2 μL 2.5 mM dNTPs, 0.8 μL forward primer (5 μM), 0.8 μL reverse primer (5 μM), 0.4 μL TransStart FastPfu DNA Polymerase, and 10 ng template DNA, and finally up to 20 μL by ddH_2_O. PCRs were performed in triplicate. The PCR products were separated on a 2% agarose gel, then extracted and purified using the AxyPrep DNA Gel Extraction Kit (Axygen Biosciences, Union City, CA, USA) according to the manufacturer’s instructions. Finally, purified products were quantified using a Quantus Fluorometer (Promega, USA).

### Illumina MiSeq sequencing and processing

The purified library was constructed as described by the Illumina library preparation protocols and then paired-end sequenced on an Illumina MiSeq PE300 platform(Illumina, San Diego, USA) according to the standard protocols of Shanghai Majorbio Bio-Pharm Technology Co. Ltd. (China).

The raw reads were quality-filtered using fastp (version 0.20.0)^74^ and merged by FLASH (version 1.2.7)^75^ using the following criteria: (1) 300 bp reads were truncated at any site receiving an average quality score < 20 over a 50 bp sliding window, and both truncated reads shorter than 50 bp and reads containing ambiguous characters were discarded; (2) only overlapping sequences longer than 10 bp were assembled, with a maximum mismatch ratio of 0.2 for the overlap region, and reads that could not be assembled were discarded; (3) samples were distinguished according to their barcoded primers, the sequence direction adjusted, and exact barcode matching was performed with a 2-nt mismatch in primer matching.

OTUs with a 97% similarity cut-off^76,77^ were clustered using UPARSE version 7.1^76^, and chimeric sequences were identified and removed. The taxonomy of each representative OTU sequence was analyzed using the RDP Classifier (version 2.2)^78^ against a 16S rRNA database (Silva v138) with a confidence threshold of 0.7.

### Statistical analysis

Species richness and biodiversity were estimated by the Chao1 estimator, Simpson diversity index, and Good’s nonparametric coverage (coverage). PCoA based on calculated Bray Curtis distances was used to explore the differences in OTU levels among rhizosphere samples in continuous cropping lily. Based on the average of the same treatment and one-way analysis of variance (ANOVA), community bar plot analysis was performed to identify significantly different phyla and orders of bacterial taxa among the groups. RDA was performed to analyze the relationships between rhizosphere soil properties and dominant bacterial orders. SEM framework was used to determine the relative importance of earthworm, OM content, available nutrients, and bacterial community on lily yields. The first component of PCA was conducted with available nutrients represented AN, AP, and AK was to simplify the SEM model. The microbial community was represented by the first component of the PCA conducted with the top ten dominant bacteria orders. ANOVA and PCA were performed using IBM SPSS 20.0 (SPSS Inc., USA).

## Supplementary Information

Supplementary Figures.

## References

[1] Li J (2019). Development, progress and future prospects in cryobiotechnology of *Lilium* spp. Plant Methods.

[2] Jin L, Zhang Y, Yan L, Guo Y, Niu L (2012). Phenolic compounds and antioxidant activity of bulb extracts of six *Lilium* species native to China. Molecules.

[3] Zhao B, Zhang J, Guo X, Wang J (2013). Microwave-assisted extraction, chemical characterization of polysaccharides from *Lilium davidii* var. *unicolor* Salisb and its antioxidant activities evaluation. Food Hydrocoll..

[4] Wu Z (2015). In vitro study of the growth, development and pathogenicity responses of *Fusarium oxysporum* to phthalic acid, an autotoxin from Lanzhou lily. World J. Microb. Biot..

[5] Wu Z (2015). Identification of autotoxins from root exudates of Lanzhou lily (*Lilium davidii* var. *unicolor*). Allelopathy J..

[6] Vaitauskienė K, Šarauskis E, Naujokienė V, Liakas V (2015). The influence of free-living nitrogen-fixing bacteria on the mechanical characteristics of different plant residues under no-till and strip-till conditions. Soil Till. Res..

[7] Doornbos RF, van Loon LC, Bakker PAHM (2012). Impact of root exudates and plant defense signaling on bacterial communities in the rhizosphere. A review. Agron. Sustain. Dev..

[8] Liu X (2020). Long-term greenhouse cucumber production alters soil bacterial community structure. J. Soil Sci. Plant Nut..

[9] Liu X (2014). Microbial community diversities and taxa abundances in soils along a seven-year gradient of potato monoculture using high throughput pyrosequencing approach. PLoS ONE.

[10] Li X, Ding C, Zhang T, Wang X (2014). Fungal pathogen accumulation at the expense of plant-beneficial fungi as a consequence of consecutive peanut monoculturing. Soil Biol. Biochem..

[11] Zhao Q (2018). Long-term coffee monoculture alters soil chemical properties and microbial communities. Sci. Rep..

[12] Shang Q (2016). Illumina-based analysis of the rhizosphere microbial communities associated with healthy and wilted Lanzhou lily (*Lilium davidii* var. *unicolor*) plants grown in the field. World J. Microb. Biot..

[13] Wu K (2014). Effects of bio-organic fertilizer plus soil amendment on the control of tobacco bacterial wilt and composition of soil bacterial communities. Biol. Fert. Soils.

[14] Baćmaga M, Wyszkowska J, Kucharski J (2018). The influence of chlorothalonil on the activity of soil microorganisms and enzymes. Ecotoxicology.

[15] Ding H (2019). Influence of chlorothalonil and carbendazim fungicides on the transformation processes of urea nitrogen and related microbial populations in soil. Environ. Sci. Pollut. R..

[16] Passari AK (2018). Biocontrol of *Fusarium* wilt of *Capsicum annuum* by rhizospheric bacteria isolated from turmeric endowed with plant growth promotion and disease suppression potential. Eur. J. Plant Pathol..

[17] Wang L (2017). Application of bioorganic fertilizer significantly increased apple yields and shaped bacterial community structure in orchard soil. Microb. Ecol..

[18] Bi Y (2018). Differential effects of two earthworm species on *Fusarium* wilt of strawberry. Appl. Soil Ecol..

[19] Zhao F (2020). Vermicompost improves microbial functions of soil with continuous tomato cropping in a greenhouse. J. Soil Sediment.

[20] Mendes R (2011). Deciphering the rhizosphere microbiome for disease-suppressive bacteria. Science.

[21] Hermans SM (2017). Bacteria as emerging indicators of soil condition. Appl. Environ. Microb..

[22] Brown ME, Chang MCY (2014). Exploring bacterial lignin degradation. Curr. Opin. Chem. Biol..

[23] Tian J, Pourcher A, Bouchez T, Gelhaye E, Peu P (2014). Occurrence of lignin degradation genotypes and phenotypes among prokaryotes. Appl. Microbiol. Biot..

[24] She S (2017). Significant relationship between soil bacterial community structure and incidence of bacterial wilt disease under continuous cropping system. Arch. Microbiol..

[25] Shen Z (2015). Soils naturally suppressive to banana *Fusarium* wilt disease harbor unique bacterial communities. Plant Soil.

[26] Feng XY (1985). The taxonomic characteristics of various genera of terrestrial earthworms in China. Chin. J. Zool..

[27] Wu WR (2008). Studies on the Germplasm Resources and Quality Estimation of Dilong (Eartheworm).

[28] Zhou L (2018). Effects of lily/maize intercropping on rhizosphere microbial community and yield of *Lilium davidii* var. *unicolor*. J. Basic Microb..

[29] Shi GY (2020). Soil fungal diversity loss and appearance of specific fungal pathogenic communities associated with the consecutive replant problem (CRP) in lily. Front. Microbiol..

[30] Blouin M (2013). A review of earthworm impact on soil function and ecosystem services. Eur. J. Soil Sci..

[31] Doran JW, Zeiss MR (2000). Soil health and sustainability: Managing the biotic component of soil quality. Appl. Soil Ecol..

[32] Kaneda S, Ohkubo S, Wagai R, Yagasaki Y (2016). Soil temperature and moisture-based estimation of rates of soil aggregate formation by the endogeic earthworm *Eisenia japonica* (Michaelsen, 1892). Biol. Fert. Soils.

[33] Bottinelli N (2010). Earthworms accelerate soil porosity turnover under watering conditions. Geoderma.

[34] Eriksen-Hamel NS, Whalen JK (2007). Impacts of earthworms on soil nutrients and plant growth in soybean and maize agroecosystems. Agric. Ecosyst. Environ..

[35] Rousk J, Brookes PC, Baath E (2010). The microbial PLFA composition as affected by pH in an arable soil. Soil Biol. Biochem..

[36] Stiles CM, Murray TD (1996). Infection of field-grown winter wheat by *cephalosporium gramineum* the effect of soil pH. Phytopathology.

[37] Weyman-Kaczmarkowa W, Pedziwilk Z (2000). The development of fungi as affected by pH and type of soil, in relation to the occurrence of bacteria and soil fungistatic activity. Microbiol. Res..

[38] Salmon S (2001). Earthworm excreta (mucus and urine) affect the distribution of springtails in forest soils. Biol. Fert. Soils.

[39] García-Montero LG, Valverde-Asenjo I, Grande-Ortíz MA, Menta C, Hernando S (2013). Impact of earthworm casts on soil pH and calcium carbonate in black truffle burns. Agroforest. Syst..

[40] Bending GD, Turner MK, Jones JE (2002). Interactions between crop residue and soil organic matter quality and the functional diversity of soil microbial communities. Soil Biol. Biochem..

[41] Wang H (2019). Effects of long-term application of organic fertilizer on improving organic matter content and retarding acidity in red soil from China. Soil Till. Res..

[42] Blouin M, Sery N, Cluzeau D, Brun JJ, Bédécarrats A (2013). Balkanized research in ecological engineering revealed by a bibliometric analysis of earthworms and ecosystem services. Environ. Manage..

[43] Wu Y, Zhang N, Wang J, Sun Z (2012). An integrated crop-vermiculture system for treating organic waste on fields. Eur. J. Soil Biol..

[44] Basker A, Macgregor AN, Kirkman JH (1992). Influence of soil ingestion by earthworms on the availability of potassium in soil: An incubation experiment. Biol. Fert. Soils.

[45] Canellas LP, Olivares FL, Okorokova-Facanha AL, Facanha AR (2002). Humic acids isolated from earthworm compost enhance root elongation, lateral root emergence, and plasma membrane H^+^-ATPase activity in maize roots. Plant Physiol..

[46] Dvorak J (2016). Sensing microorganisms in the gut triggers the immune response in *Eisenia andrei* earthworms. Dev. Comp. Immunol..

[47] Ma X, Xing M, Wang Y, Xu Z, Yang J (2016). Microbial enzyme and biomass responses: Deciphering the effects of earthworms and seasonal variation on treating excess sludge. J. Environ. Manage..

[48] Groffman PM (2015). Earthworms increase soil microbial biomass carrying capacity and nitrogen retention in northern hardwood forests. Soil Biol. Biochem..

[49] Gomez-Brandon M, Lazcano C, Lores M, Dominguez J (2010). Detritivorous earthworms modify microbial community structure and accelerate plant residue decomposition. Appl. Soil Ecol..

[50] Dempsey MA, Fisk MC, Fahey TJ (2011). Earthworms increase the ratio of bacteria to fungi in northern hardwood forest soils, primarily by eliminating the organic horizon. Soil Biol. Biochem..

[51] Wolfarth F, Schrader S, Oldenburg E, Weinert J (2011). Contribution of the endogeic earthworm species *Aporrectodea caliginosa* to the degradation of deoxynivalenol and *Fusarium* biomass in wheat straw. Mycotoxin Res..

[52] Paul BK, Lubbers IM, van Groenigen JW (2012). Residue incorporation depth is a controlling factor of earthworm-induced nitrous oxide emissions. Glob. Change Biol..

[53] Chen Y, Chang SKC, Chen J, Zhang Q, Yu H (2018). Characterization of microbial community succession during vermicomposting of medicinal herbal residues. Bioresource Technol..

[54] Tao J (2009). Effects of earthworms on soil enzyme activity in an organic residue amended rice-wheat rotation agro-ecosystem. Appl. Soil Ecol..

[55] Bertrand M (2015). Earthworm services for cropping systems. A review. Agron. Sustain. Dev..

[56] Jayasinghe BATD, Parkinson D (2009). Earthworms as the vectors of actinomycetes antagonistic to litter decomposer fungi. Appl. Soil Ecol..

[57] Fierer N, Bradford MA, Jackson RB (2007). Toward an ecological classification of soil bacteria. Ecology.

[58] Kopecky J (2011). Actinobacterial community dominated by a distinct clade in acidic soil of a waterlogged deciduous forest. Fems Microbiol. Ecol..

[59] Sun J, Zhang Q, Zhou J, Wei Q (2014). Pyrosequencing technology reveals the impact of different manure doses on the bacterial community in apple rhizosphere soil. Appl. Soil Ecol..

[60] Bull AT, Stach JEM, Ward AC, Goodfellow M (2005). Marine actinobacteria: perspectives, challenges, future directions. Anton. Leeuw. Int. J. G..

[61] Wang Q (2018). Long-term fertilization changes bacterial diversity and bacterial communities in the maize rhizosphere of Chinese Mollisols. Appl. Soil Ecol..

[62] Jones RT (2009). A comprehensive survey of soil acidobacterial diversity using pyrosequencing and clone library analyses. ISME J..

[63] Liu L (2020). *Neorhizobium lilium* sp. nov., an endophytic bacterium isolated from *Lilium pumilum* bulbs in Hebei province. Arch. Microbiol..

[64] Vessey JK (2003). Plant growth promoting rhizobacteria as biofertilizers. Plant Soil.

[65] Taylor WJ, Draughon FA (2001). *Nannocystis exedens*: A potential biocompetitive agent against *Aspergillus flavus* and *Aspergillus parasiticus*. J. Food Protect..

[66] Viswanathan R, Samiyappan R (2002). Induced systemic resistance by fluorescent pseudomonads against red rot disease of sugarcane caused by *Colletotrichum falcatum*. Crop Prot..

[67] Wolters V (2000). Invertebrate control of soil organic matter stability. Biol. Fert. Soils.

[68] Manna MC, Jha S, Ghosh PK, Achaya CL (2003). Comparative efficacy of three epigeic earthworms under different deciduous forest litters decomposition. Bioresource Technol..

[69] Felten D, Emmerling C (2009). Earthworm burrowing behaviour in 2D terraria with single-and multi-species assemblages. Biol. Fert. Soils.

[70] Wang Z (2020). Soil protist communities in burrowing and casting hotspots of different earthworm species. Soil Biol. Biochem..

[71] Danielle J, Yvan C, Daniel C (2001). Interactions between earthworm species in artificial soil cores assessed through the 3D reconstruction of the burrow systems. Geoderma.

[72] Gomes NCM (2003). Dynamics of fungal communities in bulk and maize rhizosphere soil in the tropics. Appl. Environ. Microb..

[73] Bao SD (2000). Analytical Methods of Soil Agrochemistry.

[74] Chen S, Zhou Y, Chen Y, Gu J (2018). Fastp: An ultra-fast all-in-one FASTQ preprocessor. Bioinformatics.

[75] Magoč T, Salzberg SL (2011). FLASH: Fast length adjustment of short reads to improve genome assemblies. Bioinformatics.

[76] Edgar RC (2013). UPARSE: Highly accurate OTU sequences from microbial amplicon reads. Nat. Methods.

[77] Stackebrandt E, Goebel BM (1994). Taxonomic note: A place for DNA-DNA reassociation and 16S rRNA sequence analysis in the present species definition in bacteriology. Int. J. Syst. Evol. Micr..

[78] Wang Q, Garrity GM, Tiedje JM, Cole JR (2007). Naive Bayesian classifier for rapid assignment of rRNA sequences into the new bacterial taxonomy. Appl. Environ. Microbiol..

