# Supplementary material for: Earthworm activity optimized the rhizosphere bacterial community structure and further alleviated the yield loss in continuous cropping lily (Lilium lancifolium Thunb.)
Source: Sci Rep. 2021 Oct 21;11:20840. doi: 10.1038/s41598-021-99597-y (PMC8531344; doi:10.1038/s41598-021-99597-y)
Supplement: Supplementary file 1 — Supplementary Figures. [file 41598_2021_99597_MOESM1_ESM.pdf]

Earthworm activity optimized the rhizosphere bacterial community structure and further alleviated the yield loss in continuous cropping lily (*Lilium lancifolium* Thunb.)

Yaoxiong Lu[1, 2], Peng Gao[2], Yunsheng Wang[1], Wei Li[1], Xinwei Cui[2], Jiamin Zhou[2], Fuyuan Peng[2] & Liangying Dai[1]

[1]College of Plant Protection, Hunan Agricultural University, Changsha 410128, China. [2]Institute of Agro-Environment and Ecology, Hunan Academy of Agricultural Sciences, Changsha 410125, China.

***Corresponding author:** Fuyuan Peng & Liangying Dai.

E-mail address: pengfuyuan888@163.com (for F. P.) & daily@hunau.net (for L. D.).

Journal of submission: Scientific Reports

Article type: Original research

Includes

Figure S1. The linear relationship between the incidence of leaf blight and lily yield (A), and disease index of leaf blight and lily yield (B) in 2018.

Figure S2. Relative abundances of different bacterial phyla.

Figure S3. Relative abundances of the top 10 bacterial orders.

Figure S4. Spearman correlation heatmap between the relative abundances of the top 10 bacterial orders and environmental variables.

Supplementary materials

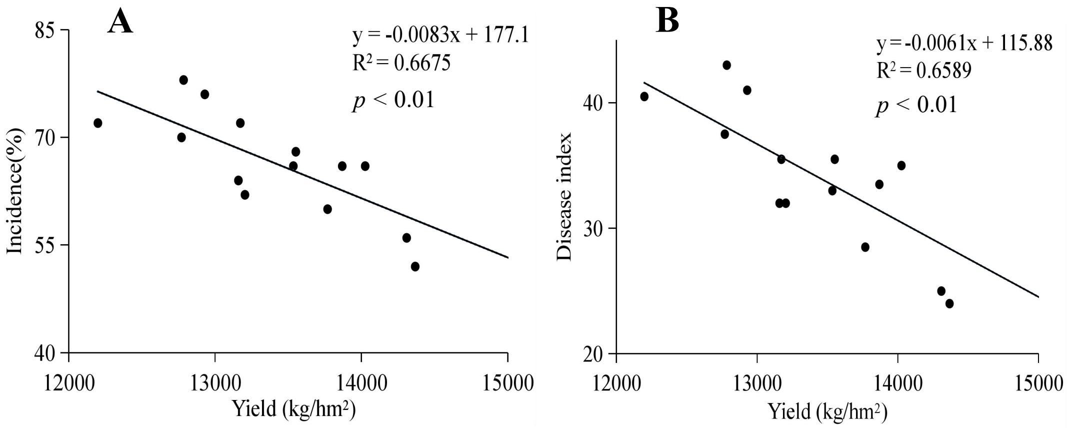

Figure S1. The linear relationship between the incidence of leaf blight and lily yield (A), and disease index of leaf blight and lily yield (B) in 2018.

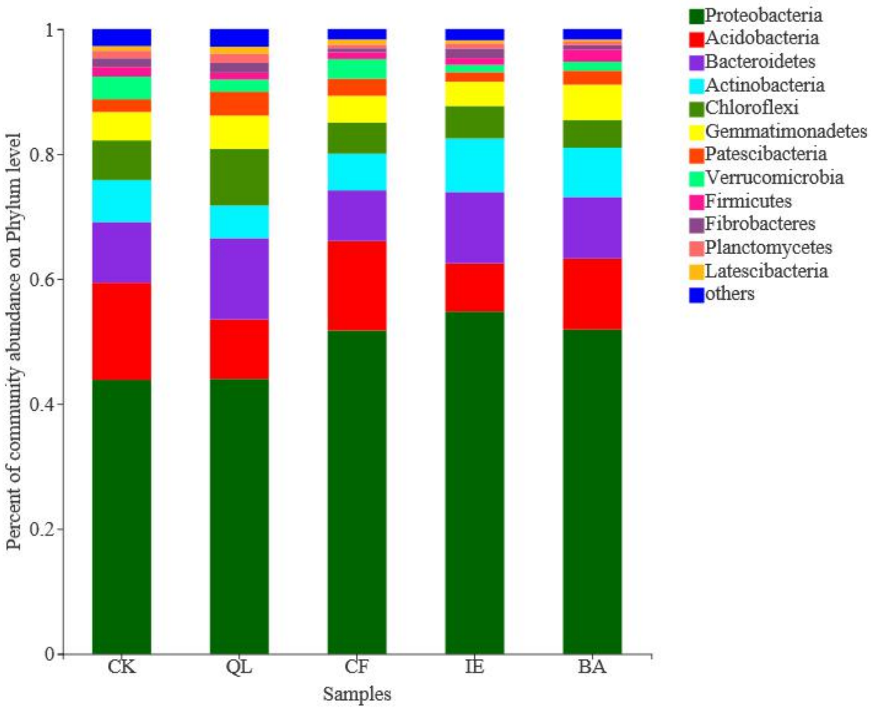

Figure S2. Relative abundances of different bacterial phyla. CK, blank control; QL, quicklime; CF, chemical fungicide; IE, inoculation with earthworms; BA, biocontrol agent.

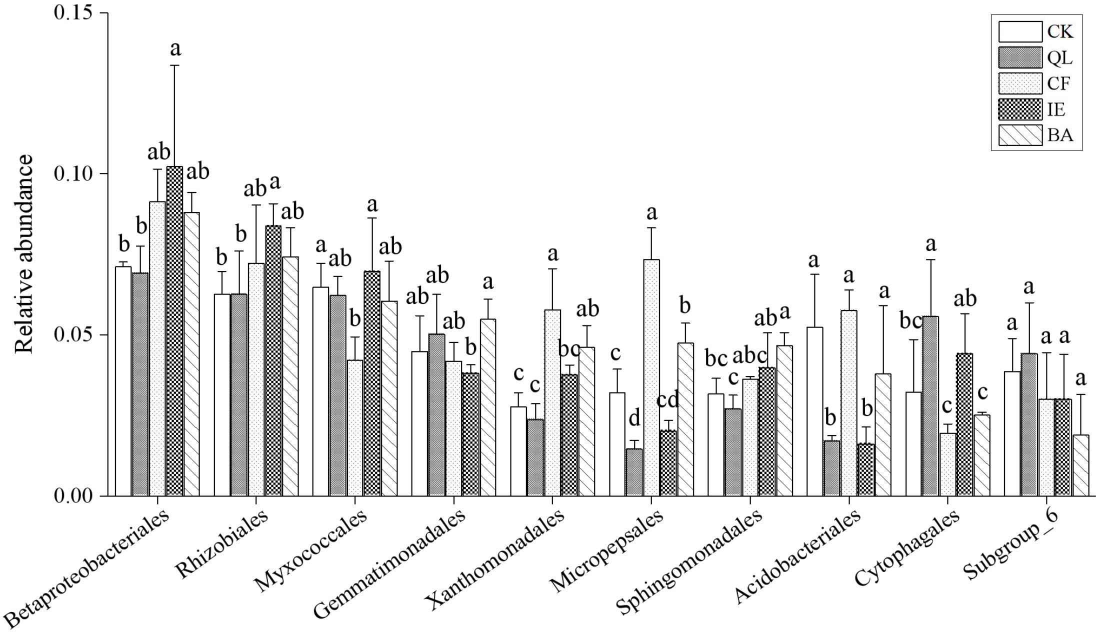

Figure S3. Relative abundances of the top 10 bacterial orders. Bars indicate standard error (n=3). Different letters above columns within the same species indicate significance at $p<0.05$ according to Duncan's test.. CK, blank control; QL, quicklime; CF, chemical fungicide; IE, inoculation with earthworms; BA: biocontrol agent.

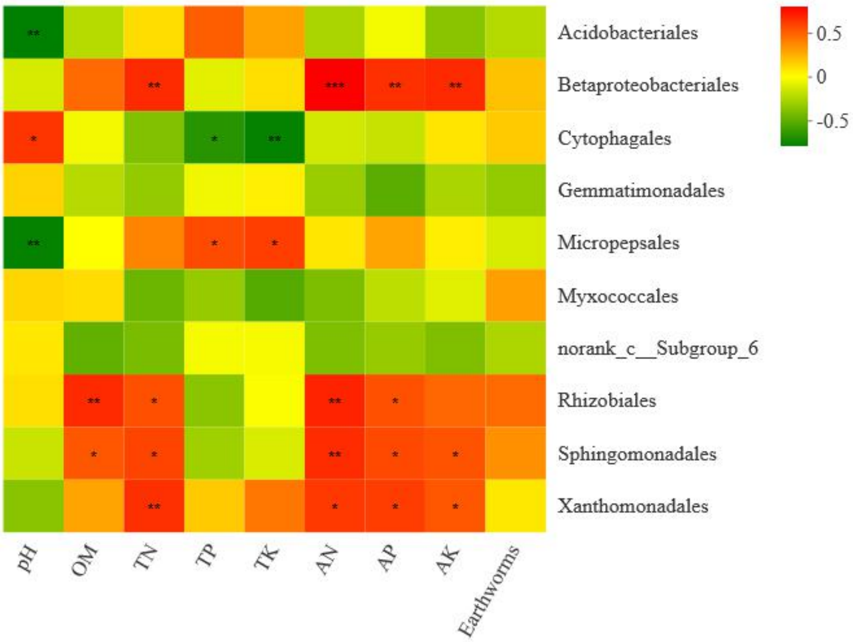

Figure S4. Spearman correlation heatmap between the relative abundances of the top 10 bacterial orders and environmental variables. pH, hydrogen ion concentration; OM, soil organic matter; TN, total nitrogen; TP, total phosphorus; TK, total potassium; AN, alkali-hydrolyzable nitrogen; AP, available phosphorus; AK, available potassium; *$p < 0.05$, **$p < 0.01$, ***$p < 0.001$.
